# Do we use antibiotics judiciously enough? A study in Accident & Emergency Department of tertiary care hospital in Pakistan

**DOI:** 10.12669/pjms.346.15542

**Published:** 2018

**Authors:** Omar Abbas Malik, Asim Saeed Khan

**Affiliations:** 1 *Omar Abbas Ahmed Malik, Student, MBBS, Dow Medical College, Dow University of Health Sciences, Karachi, Pakistan*; 2 *Asim Saeed Khan, MBBS (SMC), House Officer (2015-16 during the period of the study), Jinnah Post Graduate Medical Centre, Karachi, Pakistan*

**Keywords:** Abrasions, Antibiotics, Lacerations, Trauma

## Abstract

**Objective:**

To determine whether antibiotics are necessary for all minor wounds presenting to the Accident and Emergency Department at a tertiary care Centre in Pakistan.

**Methods:**

One hundred and five patients presenting to the Accident & Emergency Department, Jinnah Postgraduate Medical Centre, Karachi, with open wounds were included in the study and divided into two: Groups A (study) and B (control), with Group-A receiving conservative therapy. Eighty-four patients were included in the final analysis as the rest were lost to follow up. Follow up was done after one week to see how many patients had developed infection.

**Results:**

The average age of patients was 27.3 +/-9.7 years with similar baseline characteristics. From these, 51% had superficial wounds; average number of wounds was 1.63 +/-0.99, with an average length of 2.7 +/-1.6 cm. A total of 10 out of 84 patients developed infection at 7-10 days after presentation to the A&E. From these, 3 patients receiving conservative treatment (A, 10%, OR=0.107), and seven patients receiving prophylactic antibiotics (B, 12.96%, OR=0.149) developed an infection. Calculated odds ratio for increased risk of infection in Group-A = 0.72.

**Conclusion:**

A conservative approach to antibiotic prescription for minor trauma may be appropriate despite absence of strict asepsis during emergency wound care.

## INTRODUCTION

Patients commonly present to the Emergency Department after minor trauma, usually secondary to falls or other trivial accidents. Traditionally, it is felt that, due to absence of strict asepsis during wound care, as well as multiple patient factors, all patients with open wounds regardless of severity should be prescribed prophylactic antibiotics to prevent wound infection.

In contrast to this, the World Health Organization (WHO)^1^, as well as other regional organizations (e.g. IDSA^2^), employs stringent guidelines for antibiotic prophylaxis and treatment after trauma to curtail unnecessary use. These guidelines largely apply to more severe wounds that would require management in an OR, since prophylactic antimicrobials are not generally administered for superficial wounds. This is because uncontrolled use of antibiotics is potentially harmful - it exposes patients to the unnecessary risk of antibiotic associated side effects (including anaphylaxis and GI symptoms), as well as increases the risk of emergence of antibiotic resistance within local pathogens.^3^

In this study, we aimed to determine the effectiveness of conservative antibiotic prescription approach in local population.

## METHODS

### Patients

After approval from the Institutional Review Board, all hemodynamically normal patients presenting to the Accident and Emergency Department (A&E), Jinnah Postgraduate Medical Centre (JPMC), Karachi, from December 2015 to February 2016 were considered. In this period, 105 patients with minor wounds not requiring referral to other departments were enrolled following informed consent. Number, site, length, and depth of wounds, along with patient demographics, were recorded.

### Patient Exclusion Criteria

1. Patient’s age – Age < 12yr or > 60yr.
2. Immunocompromised patients (HIV, Immunosuppressive treatment, etc.).
3. Patients with Chronic Renal Failure, Diabetes, or Cirrhosis.
4. Patients with indwelling prostheses.
5. Patients with connective tissue diseases.
6. Patients currently taking antibiotics (for any reason).
7. Patients taking drugs that may interact with drugs used in this study.
8. All patients being referred to other departments (due to fractures, complicated injuries, etc.).

### Techniques

Patients that gave consent were randomized into two groups: Group-A received antibiotics as outlined below; Group-B received antibiotics for all open wounds.

### Prescription of antibiotics on discharge for

1. All contaminated and/or infected wounds^1^
2. All human bite wounds and deep animal wounds^4^
3. All wounds > 5cm in length or receiving > 7 sutures
4. All wounds > 1 cm in depth
5. Wounds older than 12 hours^1^
6. Crush injuries not treated by delayed closure^4^
7. Injuries to high-risk regions (intra-oral, on hands/feet) 6

Patients in Group-A not meeting the above criteria were discharged without an antibiotic prescription.

### Drugs used

Co-Amoxiclav, 625 mg to 1 g BID. The exact dose was left to the physician’s discretion, on the infection risk based on history, presentation, number of wounds, etc. After treatment in the ER, all the patients were instructed on wound care methods and told to change the dressing every second day.

## RESULTS

We enrolled 105 patients, 21 of whom were lost to follow-up, or did not comply with given instructions on antibiotic use. Of the remaining 84 patients (mean age 27.3 years, N=84), 30 (35.7%) had been assigned to Group-A, and 54 (64.3%) had been randomly assigned to Group-B.

Mean number of wounds was 1.6, SD+/-1.0; with a mean wound length of 2.7, SD+/-1.6 cm. Of these patients, 43 (51.2%) had superficial lacerations, 30 (35.7%) had partial thickness lacerations and 11 (13.1%) had full thickness lacerations (Table-III). A total of 10 patients developed infection at 7-10 days after presentation to the A&E (Table-IV).

**Table-I:** Classification of wound thickness.

Classification of Depth	Definition
Superficial	Through the epidermis, with intact dermis
Partial Thickness	Partially through dermis, with deeper portion still intact
Full Thickness	Through dermis, with underlying structures exposed

**Table-II:** Patients characteristics.

Group	n	Mean Age (years)	%	Males	Females
A (Study)	30	28.1±11.0	35.7	30	0
B (Control)	54	26.9±9.0	64.3	54	0

Total	84	27.3±9.7	100	84	0

**Table-III:** Wound characteristics of enrolled participants.

Group	n	Mean number of wounds	Mean wound length (cm)	Superficial	Partial thickness	Full thickness
A	30	1.7+/-1.1	3.1+/-1.9	17(56.7%)	7(23.3%)	6(20.0%)
B	54	1.5+/-1.0	2.5+/-1.3	26(48.1%)	23(42.6%)	5(9.3%)

Total	84	1.6+/-1.0	2.7+/1.6	43(51.2%)	30(35.7%)	11(13.1%)

**Table-IV:** Details of infected wounds.

Patient	1	2	3	4	5	6	7	8	9	10

Group	Study	Control	Study	Study	Control	Control	Control	Control	Control	Control
Site	Middle finger of left hand	Left wrist	Occipital region	All fingers of right hand	Right index finger	Right elbow, thigh, knee and foot	Left ear lobe	Index finger of left hand	Left thumb	Just above right ankle
Size (cm)	2.5	2.0	5.0	2.0	1.0	2.5	2.0	3.5	3.5	0.5
Depth	Full thickness	Partial thickness	Partial thickness	Partial thickness	Partial thickness	Partial thickness	Partial thickness	Full thickness	Partial thickness	Superficial
Sutures received	3	3	4	5	4	4	3	0	3	0
Dressing frequency	Daily	After two days	After 3 days	Daily	Twice daily	Alternate day	Daily	Daily	Daily	Not changed

Of the patients that developed infection, 7/10 had changed their dressing on a daily basis; 8/10 had injuries to an extremity (which is considered a high-risk region); 1/10 had a scalp laceration and 1/10 had an injury to the earlobe. Partial thickness wounds were present in 7 patients, two patients had full thickness wounds and one had a superficial wound.

From Group-A, 3/30 patients (10.0%, OR = 0.107), and from Group-B, 7/54 patients (13.0%, OR = 0.149) developed an infection. Calculated odds ratio for increased risk of infection in Group-A = 0.72.

## DISCUSSION

It is our observation that, in Pakistan, patients presenting to ER with minor lacerations, or even abrasions, due to any cause are generally prescribed antibiotics prophylactically, though we were unable to find exact statistics in the literature regarding the prevalence of this practice in the country. Studies carried out in other parts of the world show that Emergency Room physicians tend to prescribe antibiotics despite lack of evidence of their efficacy.^7^ In Pakistan, this is possibly due to the belief that the majority of population (which is impoverished) is at a greater risk of infection. There are many factors which contribute to this belief, including generally poor hygiene, wide spread malnutrition, lack of knowledge regarding proper wound care, and closure of wounds in the absence of aseptic precautions.

However studies have shown that wound closure for minor wounds need not be done in a sterile environment, as long as techniques for surgical cleanliness are used.^8^,^9^ Hygienic practices and proper wound care can be addressed at discharge and is not a suitable reason to prescribe antibiotics. International guidelines, opinions and consensus statements^1^,^10^-^12^ advise against antibiotics for minor open wounds as they have no proven benefit. Larger penetrating wounds, however, should be managed with prophylactic antibiotics. Older wounds (>12 hours), and obvious infection also warrant antibiotics. Other high-risk wounds including crush injuries and lacerations of oral mucosa may also require antibiotic therapy (as mentioned earlier). Here the question arises, “why not prescribe antibiotics for small wounds just to be safe?”

Any prescription must be given after careful analysis of the benefit-to-risk ratio.^12^ In the absence of any obvious protective benefit, the prescription of prophylactic antibiotics cannot be justified. Side effects of antibiotics range from mild GI irritation^13^ to acute kidney injury^14^, *C. difficle* colitis^15^, Stevens - Johnson syndrome/Toxic Epidermal Necrolysis^16^, and life-threatening anaphylaxis.^17^ Furthermore, uncontrolled use of antibiotics contributes to development of resistant pathogens. This is rapidly becoming a major concern worldwide. Prescription of unnecessary medication puts a further strain on non-affording patients as well. We chose to exclude patients at extremes of age, and those with co-morbids that generally predispose to infections, as we felt that this subset of patients is beyond the scope of this study.

## CONCLUSION

Judicious use of antibiotics in otherwise healthy patients with minor wounds seems to be safe and offers the benefits of decreased side effects and prevention of development of antibiotic-resistant bacteria. We believe that antibiotic prescription should be avoided in patients with minor trauma and no specific risk factors for wound infection due to the potential for great benefit not just for these patients, but also for the community at large.
